# Appropriate mowing can promote the growth of *Anabasis aphylla* through the auxin metabolism pathway

**DOI:** 10.1186/s12870-024-05204-3

**Published:** 2024-05-31

**Authors:** Ping Jiang, Peng Han, Mengyao He, Guangling Shui, Chunping Guo, Sulaiman Shah, Zixuan Wang, Haokai Wu, Jian Li, Zhenyuan Pan

**Affiliations:** 1 Agricultural College, Shihezi University, Shihezi, 832003 Xinjiang China; 2 Key Laboratory of Special Fruit and Vegetables Cultivation Physiology and Germplasm Resources Utilization, Shihezi, 832003 Xinjiang China; 3 Southern Xinjiang Research Institute, Shihezi University, Tumushuk, 843806 Xinjiang China

**Keywords:** *Anabasis aphylla*, Hormone metabolites, Metabolomics, Compensatory growth, Auxin

## Abstract

*Anabasis aphylla* (*A. aphylla*), a species of the *Amaranthaceae* family, is widely distributed in northwestern China and has high pharmacological value and ecological functions. However, the growth characteristics are poorly understood, impeding its industrial development for biopesticide development. Here, we explored the regenerative capacity of *A. aphylla*. To this end, different lengths of the secondary branches of perennial branches were mowed at the end of March before sprouting. The four treatments were no mowing (M0) and mowing 1/3, 2/3, and the entire length of the secondary branches of perennial branches (M1–M3, respectively). Next, to evaluate the compensatory growth after mowing, new assimilate branches’ related traits were recorded every 30 days, and the final biomass was recorded. The mowed plants showed a greater growth rate of assimilation branches than un-mowed plants. Additionally, with the increasing mowing degree, the growth rate and the final biomass of assimilation branches showed a decreasing trend, with the greatest growth rate and final biomass in response to M1. To evaluate the mechanism of the compensatory growth after mowing, a combination of dynamic (0, 1, 5, and 8 days after mowing) plant hormone-targeted metabolomics and transcriptomics was performed for the M0 and M1 treatment. Overall, 26 plant hormone metabolites were detected, 6 of which significantly increased after mowing compared with control: Indole-3-acetyl-L-valine methyl ester, Indole-3-carboxylic acid, Indole-3-carboxaldehyde, Gibberellin A24, Gibberellin A4, and cis (+)-12-oxo-phytodienoic acid. Additionally, 2,402 differentially expressed genes were detected between the mowed plants and controls. By combining clustering analysis based on expression trends after mowing and gene ontology analysis of each cluster, 18 genes related to auxin metabolism were identified, 6 of which were significantly related to auxin synthesis. Our findings suggest that appropriate mowing can promote *A. aphylla* growth, regulated by the auxin metabolic pathway, and lays the foundation for the development of the industrial value of *A. aphylla*.

**Supplementary Information:**

The online version contains supplementary material available at 10.1186/s12870-024-05204-3.

## Introduction

*Anabasis aphylla* (*A. aphylla*), a species of the *Amaranthaceae* family that is widely distributed in northwestern China, including in Xinjiang, Gansu, and Qinghai [1], plays a key role in the ecological balance of arid areas by preventing wind erosion and combating desertification[2, 3]. Additionally, *A. aphylla* has received increasing research attention owing to its high ecological functions and pharmacological value [4, 5]. In the assimilate branches of *A. aphylla*, toxic alkaloids were observed to be enriched, for example, *N*-methylanabasine, anabasa-mine, isonicoteine [6], which are ideal candidates for biopesticides owing to their high antibacterial, fungal insecticidal activity. However, studies on *A. aphylla* have mostly focused on their seed germination [3], ecological protection [7], and chemical constituents [8]; thus, the growth characteristics are poorly understood, impeding its industrial development for biopesticide development.

Management of these perennial semi-shrubs involves similar measures, for example, mowing [9], irrigating [10], fertilizing [11], pest control [12], plowing and weeding [13], and frost prevention [14]. Mowing is a commonly used growth control method in agricultural production [15]. Proper mowing can stimulate plant growth and branching [16] and increase leaf area and photosynthetic efficiency, improving plant yield and quality [17, 18]. Compensatory growth, a maladaptive compensatory mechanism that reduces mowing stress [19–21], is an adaptive strategy for survival, reproduction, and growth [22]. Therefore, studying plant compensatory growth mechanisms is essential to understanding plant growth regulation and resource utilization efficiency. Increasing evidence is showing that multiple hormones, including cytokinins, auxins, and gibberellins, participate in plant compensatory growth after mowing [23–26].

The aforementioned studies have demonstrated that transcriptomics and metabolomics are high-throughput technologies used widely across life sciences [27–29]. In plant science, transcriptome and metabolome techniques are used to study the mechanisms through which plants adapt to environmental changes [27, 30, 31]. For example, mechanical damage may induce plant responses that lead to changes in gene transcription [32, 33] and material metabolism [34, 35]. These reactions minimize plant cell damage and mitigate the negative effects of mechanical damage to plants [36–38]. These findings highlight the crucial regulatory role of RNA [39, 40], as well as the essential roles of energy [41], metabolism [42], and hormones [36, 37] in the repair and growth of damaged tissues. Simultaneous transcriptome and metabolome analyses can interconnect information regarding gene expression and metabolic levels, unveiling the interactions and regulatory networks between these processes [43, 44]. This comprehensive approach can broaden our understanding of the mechanisms governing plant growth and repair after mechanical damage and highlight the synergistic interactions among RNA, metabolites, and hormones in these processes [45, 46].

In this study, we aimed to explore the regenerative capacity of *A. aphylla* as follows: at the end of March, different lengths of the secondary branches of perennial branches were mowed; subsequently, every 30 days, the new assimilate branches’ related traits were recorded; finally, to evaluate the mechanism of the compensatory growth after mowing, a combination of dynamic plant hormone-targeted metabolomics and transcriptomics was performed. This study provides novel insights into increasing the number of perennial branches of *A. aphylla*, laying the foundation for the development of the industrial value of *A. aphylla* for biopesticide.

## Materials and methods

### Plant material

The experiment was conducted at the Forest Management Station situated on the southwestern edge of the Gurbantunggut Desert (45°27′N, 85°0′E) in Karamay, Xinjiang, China, in 2021. The experimental materials were naturally growing *A. aphylla* semi-shrubs.

### Experimental treatment

Before sprouting in the spring, treatments were implemented at four mowing severities: (i) no mowing (M0); (ii) less-mowed (M1), 1/3 the length of the secondary branches of perennial branches were mowed; (iii) middle-mowed (M2), 2/3 the length of the secondary branches of perennial branches were mowed; and (iv) excessive-mowed (M3), for which, all secondary branches of perennial branches were mowed. The experimental design used a completely randomized pattern and three replicates for each treatment. Each treatment contained 30 relatively uniform plants.

### Phenotypic investigation

For phenotypic analysis, after the material treatment on March 25, 2021, the length and basal diameter of assimilation branches on the plants were counted at 30-day intervals. After 150 days, we calculated the biomass of assimilation branches by mowing all new assimilation branches of the year.

### Plant collection and tissue sample preparation

Before sprouting in the spring, perennial branch tips were collected from the M1 and M0 treatment groups at days 0, 1, 5, and 8, using a clean, sharp razor blade, after the former was mowed. Three biological repeats were performed during each period. The collected tissue samples were immediately frozen in liquid nitrogen and stored at -80 °C in a freezer upon return to the laboratory. The collection of plant materials complied with relevant institutional, national, and international guidelines and legislation.

### Detection and analysis of plant hormone-targeted metabolites

This experiment employed the UHPLC-MRM-MS/MS method [47] to detect and analyze a total of 88 plant hormone-targeted metabolites from 24 tissue samples. Qualitative and quantitative results for each sample were obtained using mass-spectrum data analysis. Data were dispersion-normalized for each metabolite, and metabolites with missing values were removed, yielding a relative quantification of each metabolite. Principal component analysis and patterns of metabolite accumulation were also analyzed.

### RNA isolation and detection

Total RNA was isolated from the aforementioned samples by using an RNA extraction kit (RN40; Aidlab Biotechnologies, China) per the manufacturer’s instructions. RNA purity and concentration were determined using NanoDrop^TM^ 2000 (Thermo Fisher Scientific,USA). RNA integrity was assessed using an Agient2100, LabChip GX (Platinum, Model Platinum Elmer LabChip GX, USA).

### PacBio and Illumina Library construction and sequencing

High-quality RNA from stem tip tissues of 24 assimilated shoots, obtained from the mowed and control plants, was mixed in equal amounts. The mixed product was subjected to damage repair, end repair, and ligation by using the SMRTbell Template Prep Kit, resulting in a long-read transcriptome library. The qualified library was combined with primers and polymerase by using the PacBio Binding Kit (PacBio, USA). The final reaction product was purified using AMpure PB Beads (PacBio) and sequenced using a PacBio Sequel II (PacBio) sequencing instrument.

A total of 1 μg RNA per sample was used as the input material to generate Illumina sequencing libraries by using the VAHTS Universal V6 RNA-seq Library Prep Kit for Illumina (New England Biolabs, USA). Index codes were added to attribute sequences to each sample. The quality of each library was then checked using the Qsep-400 (BiOptic Inc., Taiwan) method. The Illumina Novaseq 6000 platform (Illumina, USA) was used to sequence the qualified library.

### Transcriptome assembly and gene functional annotation

The raw reads were processed into circular consensus (CCS) reads using an adaptor. Next, full-length, non-chimeric transcripts were identified by searching for the polyA tail signal and the 5' and 3' cDNA primers in CCS. First, the full-length sequences from the same transcript were clustered; next, similar full-length sequences were clustered to obtain a consistent sequence. Consistent sequences were corrected to obtain high-quality sequences for subsequent analyses. Iso-Seq high-quality FL transcripts were used to remove redundancies by using a cluster database at high identity with tolerance. Non-redundant transcripts, measured using full-length transcriptome sequencing, were used as references for sequence alignment and subsequent analyses.

Gene function was annotated based on the following databases: NCBI non-redundant protein sequences (NR), protein family (Pfam), Clusters of Orthologous Groups (COG), Eukaryotic Orthologous Groups (KOG), a manually annotated and reviewed protein sequence database (Swiss-Prot), the Kyoto Encyclopedia of Genes and Genomes (KEGG), and gene ontology (GO).

### Differential gene expression analysis

The generated final Clean Illumina sequencing data were mapped to non-redundant transcripts obtained using full-length transcriptome sequencing. The expression level of each gene was calculated using the software RSEM and converted into fragments per kilobase per million fragments based on the read counts.

To identify differentially expressed transcripts at different developmental stages of *A. aphylla* after mowing, we compared T1 and CK1, T5, and CK5, and T8 and CK8. The selection of differentially expressed genes (DEGs) was based on specific criteria. Subsequently, the DEGs were subjected to Mfuzz [48] clustering analysis to examine their expression patterns over time. During this analysis, we focused on clusters that exhibited relatively stable transcript levels at different time points in the natural assimilation branches and similar or opposite patterns as target metabolites in the mowed assimilation branches, which were considered key candidate clusters. GO functional enrichment analysis was performed for genes in each module. Plant hormone-related genes and their corresponding transcription factors were selected for further analysis.

### Screening hub genes based on correlation analysis

To reveal the association between key genes and target metabolites, we used Spearman’s correlation coefficient for correlation analysis between gene expression and metabolic abundance. Finally, hub genes were further filtered based on absolute correlation (> 0.7) and adjusted *P* value (< 0.01).

### Data analysis

Data processing and management were conducted using Microsoft Excel (Microsoft Office Excel 2019, Microsoft Corporation) and the R statistical language (version 4.3.2). Phenotypic data analysis and visualization were executed using the R software package ggplot2 [49], leveraging its basic functionalities within the RStudio integrated development environment (RStudio, Boston, USA). Differential expression analysis was performed using the limma package [50] in R, with the selection of DEGs based on specific criteria: a false discovery rate < 0.01, fold change (FC) ≥ 2, and adjusted *P value* < 0.05. Visualization of the GO functional enrichment analysis outcomes was achieved in a word cloud created using the R package wordCloud [51]. Additionally, the study incorporated the cluster database at high identity with tolerance tool for the clustering of high-similarity sequences with an identity > 0.99. The correlation plot between key genes and target metabolites was generated employing Cytoscape (version 3.10.1, Cytoscape Consortium).

## Results

### Phenotypic analysis of compensatory growth of *A. aphylla*

**Fig. 1:**
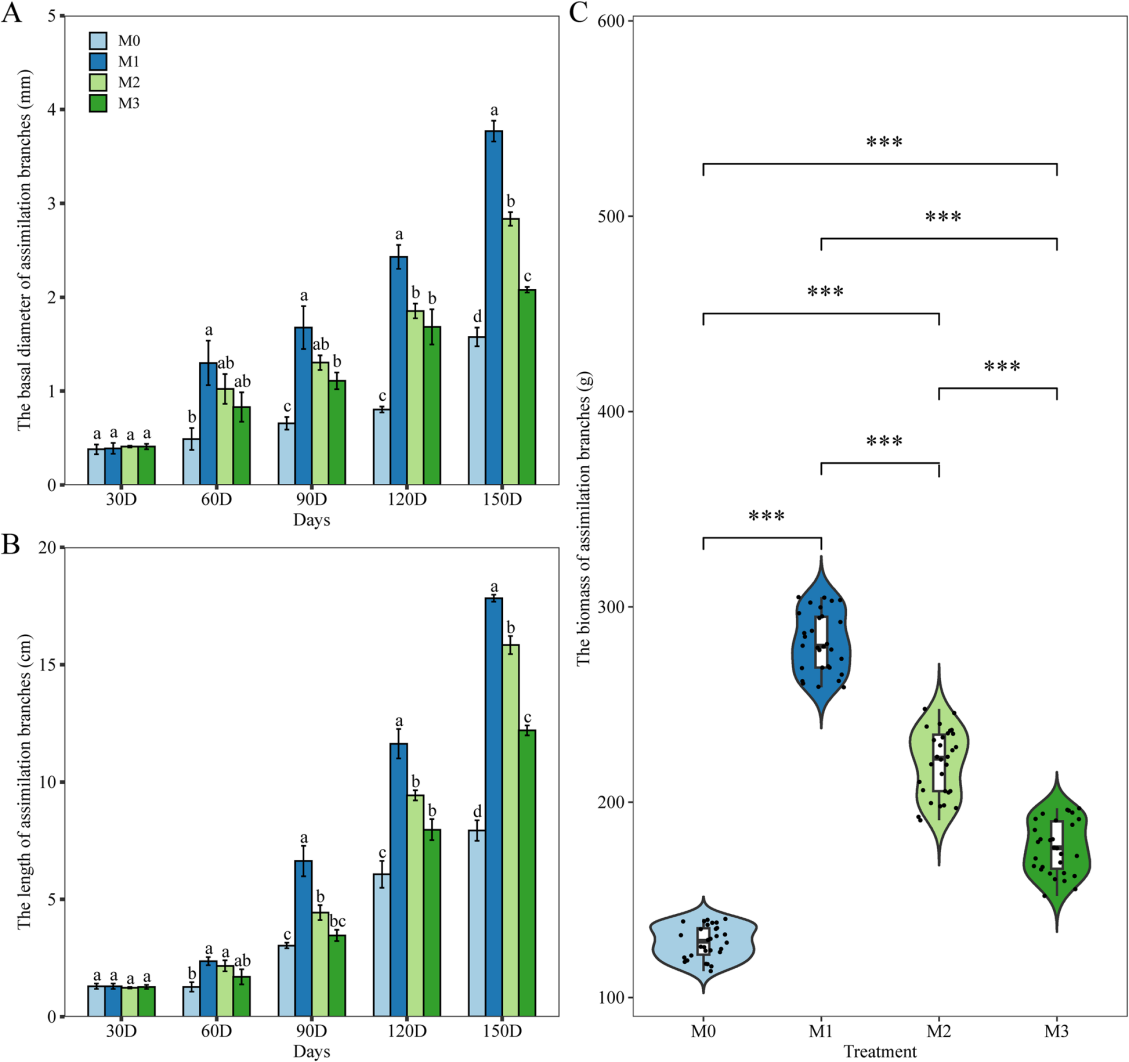
Impact of mowing treatments on growth and development of assimilation branches in *A. aphylla*. Note: **A**) base diameter of assimilation branches; **B**) length of assimilation branches; **C**) biomass of assimilation branches. Different lowercase letters indicate significant differences (*P* < 0.05); *** indicates significant correlation at 0.001 level

For the exploration of the regenerative capacity of *A. aphylla*, treatments were performed at four mowing degrees before sprouting in the spring, using uniform plants (Supplementary figure S1). To compare the impact of the treatments, we used a global ANOVA test. In *A. aphylla*, the mowed plants showed a greater basal diameter (Fig. 1A) and longer branches (Fig. 1B) of assimilation branches than un-mowed plants from 60 days after treatment, especially in the latter stages. Additionally, M1 resulted in significantly (*P* < 0.05) thicker basal diameter and longer branches of assimilation branches than the other two treatments. Finally, the biomasses of assimilation branches of the four treatments at 150 days were measured, and the biomass of assimilation branches was greater for the mowed than un-mowed plants (Fig. 1C, *P* < 0.001). Notably, with the increasing mowing degree, the biomass of assimilation branches showed a decreasing trend. In summary, M1 (i.e., mowing 1/3 the length of the secondary branches of perennial branches mowed) stimulated *A. aphylla* to strengthen its compensatory growth effect and produce the maximum biomass of assimilation branches; therefore, we used M1 treatment to uncover the mechanism of compensatory growth.

### Phytohormone-targeted metabolite analysis of assimilation branches in *A. aphylla* after mowing

**Fig. 2:**
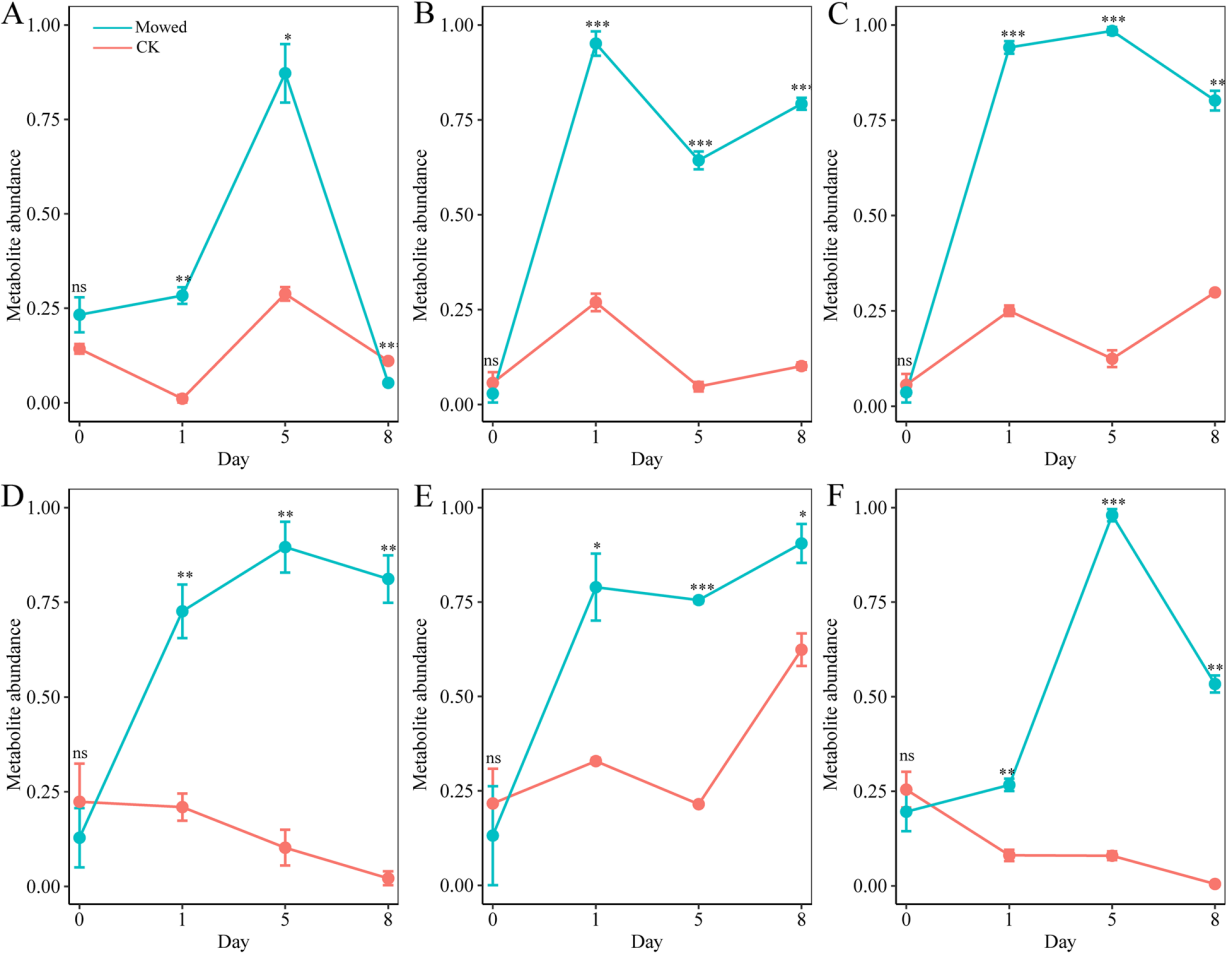
Analysis of target metabolite accumulation patterns of **A**) Indole-3-acetyl-L-valine methyl ester, **B**) Indole-3-carboxylic acid, **C**) Indole-3-carboxaldehyde, **D**) Gibberellin A24, **E**) Gibberellin A4, and **F**) cis (+)-12-Oxophytodienoic acid. Note: *, **, and *** indicate significance at *P* < 0.05, *P* < 0.01, and *P* < 0.001, respectively. The vertical line represents the standard deviation

Phytohormones play important roles in plant acclimation and the repair of mechanical injuries. To determine whether phytohormones are involved in the regulation of compensatory growth in *A. aphylla*, we performed phytohormone-targeted metabolite analysis by using tissue samples from the assimilation branches of leafless horsetail after the cutting treatment at specific time intervals (0, 1, 5, and 8 s) using the UHPLC-MRM-MS/MS method; assimilation branches without mowing were used as controls. Twenty-six metabolites were identified: nine indole derivatives, five cytokinin derivatives and related compounds, three gibberellin derivatives, five jasmonate derivatives, and four other metabolites. Principal component analysis (Supplementary figure S2) based on metabolite levels revealed that the 24 tissue samples were separated into 2 distinct clusters: mowing samples and controls. Additionally, under mowing conditions, the metabolite accumulation patterns in the assimilation branches exhibited a more pronounced spatial separation than that under the controls (Supplementary figure S2). Moreover, the differential accumulated metabolite analysis identified 16, 19, and 22 differential accumulated metabolites on days 1, 5, and 8, respectively. The analysis of the metabolite accumulation patterns (Supplementary figure S3) demonstrated that six metabolites were significantly upregulated compared with those of the natural assimilation branches: Indole-3-acetyl-L-valine methyl ester (IAA-Vel-me; Fig. 2A), Indole-3-carboxylic acid (ICA; Fig. 2B), Indole-3-carboxaldehyde (I3C; Fig. 2C), Gibberellin A24 (Fig. 2D), Gibberellin A4 (Fig. 2E), and cis (+)-12-oxo-phytodienoic acid (OPDA; Fig. 2F). Among the six metabolites, IAA-Vel-me is the precursor form of IAA [52, 53], and ICA and I3C are the metabolite products of IAA [54, 55]. Gibberellin A4 is an active gibberellin in plants [56], and Gibberellin A24 is a direct metabolite of Gibberellin A1[57]. OPDA serves as a biosynthetic precursor of jasmonic acid [58].

### RNA sequencing, de novo assembly, and functional annotation

To identify the key genes involved in the compensatory growth of *A. aphylla*, we sequenced RNA from 24 mixed samples of assimilation branches at four time points. A total of 267,802,289 bp of clean data containing 129,314 CCS reads was obtained from the PacBio sequencing platform, and the mean read length of the CCS was 2070 bp (Supplementary table S1). By detecting the positional relationships of the inserted sequences, we obtained 111,432 full-length non-chimeric reads, accounting for 86.17% of the total number of CCS sequences (Supplementary table S2). Similar sequences were clustered in the IsoSeq module of SMRTLink, and each cluster represented a consensus isoform. A total of 63,353 consensus sequences were clustered with a mean read length of 1,661 bp, including 36,350 high-quality isoforms and three low-quality isoforms. Consensus sequences were polished using Quiver to obtain 63,350 high-quality sequences (Supplementary table S3). Finally, low-quality consensus sequences were corrected using Illumina short reads. After removing redundant sequences, a total of 48,763 transcript sequences were obtained.

**Fig. 3:**
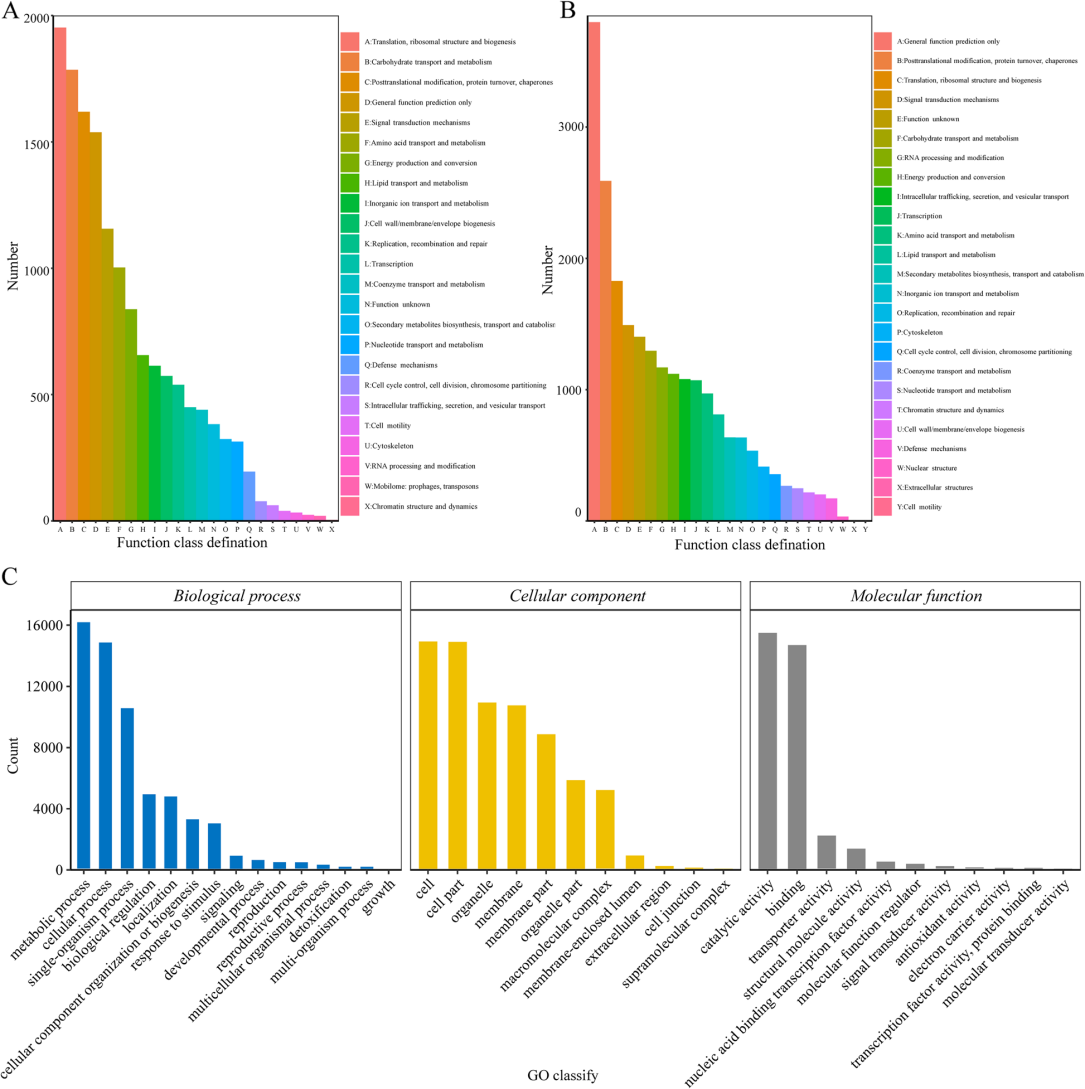
Gene functional annotation and histograms of Clusters of Orthologous Groups (COG). **A** eukaryotic orthologous groups (KOG); **B**) gene ontology (GO); and **C**) functional annotations

For the 48,763 transcripts, the COG, KOG, GO, KEGG, and NR databases were used as references for functional annotation. A total of 40,893 unigenes were successfully annotated, including 16,431 (40.18%) by COG, 24,800 (60.65%) by KOG, 29,595 (72.37%) by GO, 18,160 (44.41%) by KEGG, and 40,663 (99.42%) by NR databases (Supplementary table S4). Moreover, the 16,431 unigenes annotated by COG were subdivided into 24 COG categories (Fig. 3A), among which, the cluster “Translation, ribosomal structure and biogenesis” was the largest group (1952 unigenes), followed by “Carbohydrate transport and metabolism” (1785 unigenes). The 24,800 unigenes annotated by KOG were classified into 25 KOG categories (Fig. 3B), among which, the largest cluster was “General function prediction only” (3,799 unigenes), followed by “Posttranslational modification, protein turnover, chaperones” (2,590 unigenes). A total of 29,595 GO annotated unigenes were distributed under three major GO categories: biological processes, cellular components (CC), and molecular functions (MF) (Fig. 3C). For biological processes, the metabolic process (16,267) and cellular process (14,946) were the most significantly enriched terms; for CC, cell (15,012) and cell part (14,984) were the most significantly enriched terms; and for molecular function, binding (14,780) and catalytic activity (15,579) were the most significantly enriched terms. Functional annotation information of the assemblies included unigene protein and COG functional categories. KEGG pathway and enrichment analysis showed that 18,160 unigenes were significantly assigned to 129 enriched pathways. The top five pathways, from smallest to largest gene number, were “Carbon metabolism,” “Ribosome,” “Biosynthesis of amino acids,” “Protein processing in endoplasmic reticulum,” and “Spliceosome,” wherein 936, 839, 715, 641, and 609 related genes were enriched in these pathways, respectively (Supplementary table S5). Finally, the annotated genes in the NR database were aligned with *Chenopodium quinoa* (22.85%) and *Beta vulgaris* (21.01%) (Supplementary figure S4). Similarly, the Pfam and Swiss-Port databases were used for annotation, as the supplementary information.

### Analysis of differentially expressed genes (DEGs)

**Fig. 4:**
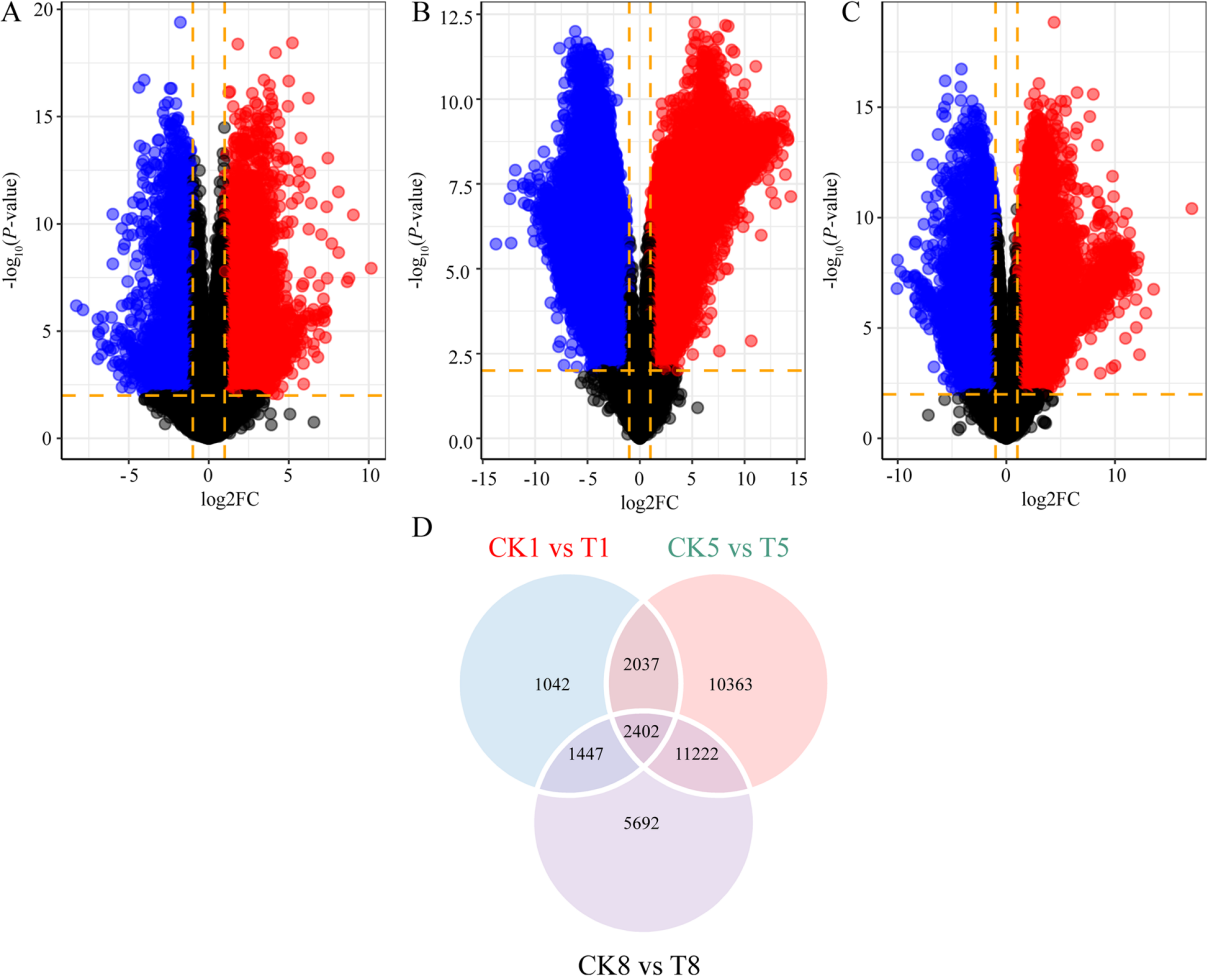
Comparative transcriptomics analysis between assimilation branches of mowed and natural plants. Note: Assimilation branches of mowed and natural at days 0, 1, 5, and 8 (renamed T0, T1, T5, T8, and CK0, CK1, CK5, and CK8, respectively) were used for second-generation sequencing. In A–C, blue dots represent downregulated differentially expressed genes, red dots represent upregulated differentially expressed genes, and black dots represent non-differentially expressed genes. **A** Differentially expressed transcript volcano map of CK1 versus T1; **B**) differentially expressed transcript volcano map of CK5 versus T8; **C**) differentially expressed transcript volcano map of CK8 versus T8; **D**) Venn diagram showing the number of unshared and shared DEGs through paired comparison

Next-generation sequencing yielded a total of 156,663,187,812 bp clean data from 24 sequencing libraries (Supplementary Table S6), including three biological replicates of mowed and natural assimilation branches at four time points. Sequence alignment between clean reads obtained by next-generation sequencing and non-redundant transcripts measured 30.94–50.83% uniquely mapped reads, 11.47–8.13% multiple aligned reads, and 0.04–21.58% too many multiple aligned reads, indicating that the transcriptome data were used efficiently in this study (Supplementary table S7). With FC ≥ 1 and false discovery rate < 0.01 as screening conditions, 6,928 (3,705 up- regulated, and 3,223 down-regulated; Fig. 4A), 26,024 (3,705 up- regulated, and 3,223 down-regulated; Fig. 4B), and 20,793 (3,705 up- regulated, and 3,223 down-regulated; Fig. 4C) DEGs were identified in the assimilation branches between mowed and natural conditions at days 1, 5, and 8, respectively. The three groups of DEGs were intersected to screen 2,402 genes (Fig. 4D) that were simultaneously differentially expressed.

### Identification of the hub genes

**Fig. 5:**
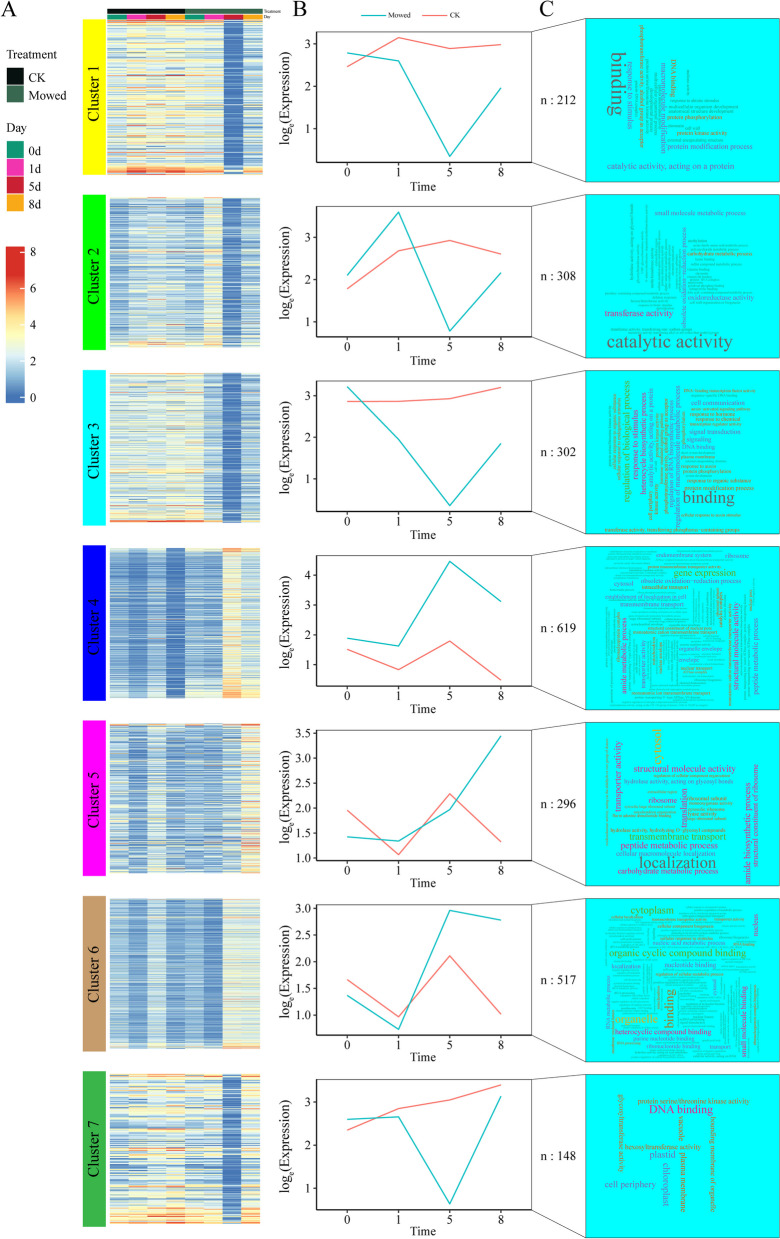
Classification and annotation of differentially expressed genes. Note: **A**) Mfuzz analysis of the 2,402 DEGs identified from the Venn diagram in Fig. 4. Seven clusters were identified based on the expression profiles of the DEGs, and heatmaps were generated for gene expression based on fragments per kilobase per million fragments; **B**) Line plots showing the transcription trends of seven gene clusters from hierarchical clustering and the number of genes in each cluster. Natural- and mowed-type transcription trends are represented by solid red and blue lines, respectively. **C**) GO enrichment word cloud for each cluster

The DEGs were divided into seven clusters by using Mfuzz cluster analysis based on the expression profiles of these genes (Fig. 5A). A total of 212, 308, 302, 619, 296, 517, and 148 DEGs were incorporated into clusters 1–7, respectively. Temporal trends in gene expression were further examined using best-fit lines generated using a standard linear model (Fig. 5B). Regarding the mowed plants, modules 1, 3, and 7 exhibited comparable patterns, where gene expression was consistently downregulated on days 1 and 5, followed by an upregulation trend on day 8; the control showed a relatively stable trend. Modules 4 and 6 showed a similar trend between the mowed and control plants, showing a decrease on day 1, followed by upregulation on day 5 and downregulation on day 8. Module 5 showed a slight decrease in gene expression on day 1 and a continuous increase in expression on days 5 and 8; the expression of the control showed a large fluctuation. Simultaneously, we performed GO enrichment analysis using the genes individually assigned to each of these modules (Fig. 5C). Notably, only genes in the cyan module were enriched in hormone regulatory pathways, such as auxin-activated signaling pathways and cellular responses to auxin stimuli. Subsequently, based on the semantics of the GO annotations (GO: 0009733, 0071365, and 0009734), 18 genes related to auxins in the cyan module were identified as key genes (Supplementary table S8).

**Fig. 6:**
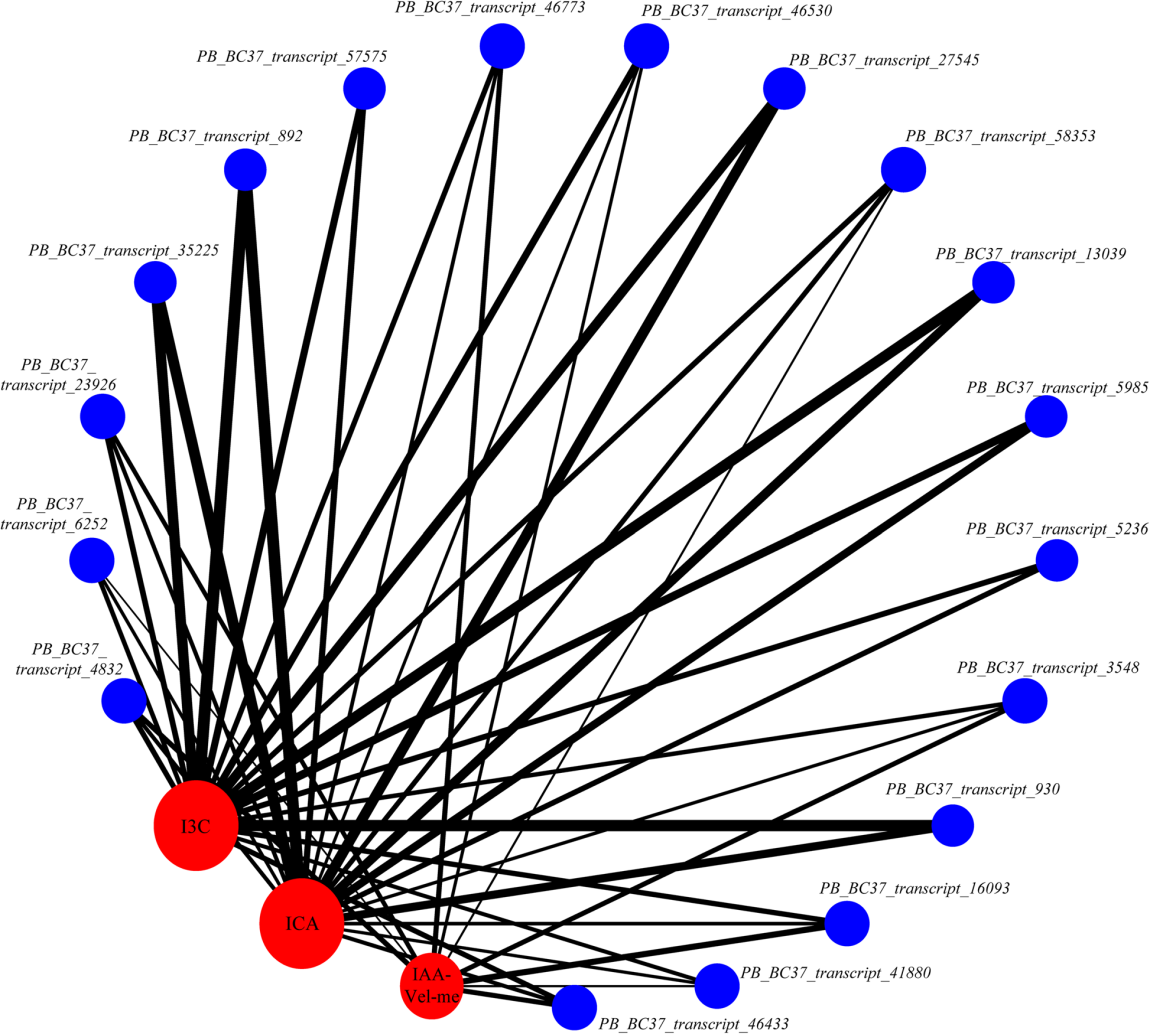
Correlation analysis of target metabolites and key genes. Note: The line connecting two parameters indicates significant correlations; line thickness represents the absolute magnitude of the correlation coefficient. The black line represents negative correlations. The circles represent nodes with several connections: red circles represent target metabolites, and blue circles represent key genes

To identify the relationship between key genes and auxin derivatives, we conducted a correlation analysis between key genes and auxin derivatives. As shown in Fig. 6, there was a significant correlation between the metabolic abundance of ICA and I3C and the expression of the 18 key genes. ICA and I3C showed strong negative correlations with the expression of the six key genes (absolute value of the correlation coefficient > 0.7, *P* < 0.01): *PB_BC37_transcript_892, PB_BC37_transcript_930, PB_BC37_transcript_5985, PB_BC37_transcript_13039, PB_BC37_transcript_27545,* and *PB_BC37_transcript_35225.* Among them, three genes (*PB-BC37_transcript_5985, PB-BC37_transcript_13039,* and *PB-BC37_transcript_35225*) belong to the Auxin/IAA (AUX/IAA) family of genes: two genes (*PB-BC37_transcript_892*, and *PB-BC37_transcript_930*) are auxin transcription factors containing B3 DNA-binding domains (belonging to the B3 superfamily of transcription factors, which also includes the AUX/IAA family); and one gene (*PB_BC37_transcript_27545*) is a membrane transport protein. Therefore, the six key genes were considered hub genes that regulate auxin metabolism (Supplementary table S9).

## Discussion

### Proper mowing promotes the growth of *A. aphylla*.

After mowing, the lateral buds were exported to the assimilation branches [59]. This phenomenon may be caused by the breaking of the apical dominance, which promotes the biomass of the plant [60]. In this study, mowed plants showed a greater growth rate of assimilation branches than un-mowed plants. Additionally, with the increasing mowing degree, the growth rate and the final biomass of assimilation branches showed a decreasing trend, with M1 resulting in the greatest growth rate and final biomass. Our results are consistent with those in the literature for other plants [60–62].

### Auxin and gibberellin metabolic pathways are key to the post-mow compensatory growth process of *A. aphylla*.

Shoot branching is another complex growth regulatory process [63, 64]. In this study, we focused on the roles of auxin and gibberellin in post-mow compensatory growth. Shoot meristems influence each other’s growth, a phenomenon particularly evident in apical dominance [65]. Auxin maintains apical dominance, inhibiting the outgrowth of lateral buds [62, 66]. When the apical meristem is cut, apical dominance is suppressed, triggering lateral bud development [62]. Studies have shown that auxins tend to control cytokinin biosynthesis to regulate lateral bud elongation for a short time [23]. When the growth advantage of lateral buds is established, the auxin content in the lateral buds increases, further promoting the growth of lateral buds [67], which also explains the increased abundances of IAA-Vel-me, ICA, and I3C. Additionally, gibberellin is a group of key hormones that regulate many aspects of plant growth and development [68]. Specifically, gibberellin is a positive regulator of shoot branching in woody plants [24]; thus, a high concentration of GA increases the number of stimulated lateral buds [24]. This conclusion is consistent with our results.

### Hub genes are the key genes involved in auxin regulation

Gene transcription is a signal transduction switch that directly regulates plant growth and development [69, 70]. In this study, we identified six hub genes involved in the post-mow compensatory growth process, three of which were AUX/IAA family members. The AUX/IAA gene family functions as a repressor, working in conjunction with the receptor (F-box protein) and transcriptional activator, auxin response factor (ARF), to regulate auxin perception and the expression of auxin-regulated genes [71–73]. Auxin-mediated transcriptional regulation is exclusively dependent on the function of AUX/IAA [74]. AUX/IAA family proteins bind to ARFs, inhibiting them and preventing the expression of auxin-responsive genes [75]. In the presence of elevated auxin levels, ubiquitination of AUX/IAA proteins typically transforms inhibitory ARF-AUX/IAA complexes into activated ARF complexes [76], which promotes the upregulation of auxin-related genes [77]. The decreased levels of AUX/IAA transcripts create a feedback mechanism that counteracts the increased stability of AUX/IAA [78]. That is, when the expression of the AUX/IAA family members is downregulated, auxin synthesis is promoted, serving as a negative feedback regulator of auxin signaling. The down-regulated expression of the three AUX/IAA family genes in our study promoted the production of IAA and subsequently enhanced the compensatory growth of horsetails.

## Conclusions

In summary, the secondary branch mowing of *A. aphylla* in the spring significantly accelerated growth and increased biomass, especially the treatment of mowing 1/3 the length of the secondary branches, which is regulated by auxin metabolic pathways. Additionally, the hub genes involved in the auxin metabolic pathways, which encoded AUX/IAA family proteins, were identified. Together, these findings can be used to inform *A. aphylla* cultivation and management strategies, which lay the foundation for the development of the industrial value of *A. aphylla*. A limitation of this study is that no data were collected on in vitro auxin treatment. In further research, we plan to study the effect of in vitro auxin spray on the compensatory growth of *A. aphylla* and elucidate the functions of candidate genes related to auxin metabolism.

### Supplementary Information

Supplementary Materials 1.

## Data Availability

The sequenced raw reads generated in this study have been submitted to the China National Center for Bioinformation (CNCB) with BioProject ID: PRJCA025227.
