# Supplementary material for: Appropriate mowing can promote the growth of Anabasis aphylla through the auxin metabolism pathway
Source: BMC Plant Biol. 2024 May 31;24:482. doi: 10.1186/s12870-024-05204-3 (PMC11141038; doi:10.1186/s12870-024-05204-3)
Supplement: Supplementary file 1 — Supplementary Materials 1. [file 12870_2024_5204_MOESM1_ESM.docx]

**Supplementary Table**

Supplementary table S1 Statistics of CCS data output of third generation sequencing.

| **Samples** | **cDNA size** | **CCS Number** | **Read Bases of CCS** | **Mean Read Length of CCS** | **Mean Number of Passes** |
| --- | --- | --- | --- | --- | --- |
| PB-BC37 | 1-6K | 129,314 | 267,802,289 | 2,070 | 80 |
| PB-BC37 | All | 129,314 | 267,802,289 | 2,070 | 80 |

Note: Samples: sample number;

CDNA Size: the size of the inserted fragment (including primer, polyA tail and cdna) when building the library;

CCS number: the number of CCS sequences;

Read bases of CCS: the total number of bases in CCS;

Mean read length of CCS: average length of CCS sequence;

Mean number of passes: the average sequencing depth (passes) of the sequences in all ZMWs in the cell.

Supplementary table S2 Full-length sequence data statistics.

| **Samples** | **Number of ccs** | **Number of undesired primers reads** | **Number of filtered short reads** | **Number of full-length non-chimeric reads** | **Full-length non-chimeric percentage (FLNC%)** |
| --- | --- | --- | --- | --- | --- |
| PB-BC37 | 129,314 | 7,833 | 0 | 111,432 | 86.17% |

Note : Samples: sample number;

Number of ccs: number of CCS sequence;

Number of undesired primers reads: number of CCS without correct primers;

Number of filtered short reads: indicates the number of filtered CCS <50bp.

Number of full-length non-chimeric reads: number of full-length non-chimeric reads;

Full-length non-chimeric percentage (FLNC%): percentage of full-length non-chimeric sequences to CCS sequences.

Supplementary table S3 Statistical results of high- and low-quality transcripts obtained using different database lengths.

| **Samples** | **Number of consensus isoforms** | **Average consensus isoforms read length** | **Number of polished high-quality isoforms** | **Number of polished low-quality isoforms** | **Percent of polished high-quality isoforms (%)** |
| --- | --- | --- | --- | --- | --- |
| PB-BC37 | 63,353 | 1,661 | 63,350 | 3 | 100.00% |

Note: Samples: sample number;

Number of consensus isoforms: the consistent sequence obtained by clustering;

Average consensus isoforms length: consistent sequence length;

Number of high-quality isoforms: the number of high-quality (accuracy > 0.99) transcripts in the consistent sequence;

Number of low-quality isoforms: the number of low-quality (accuracy < 0.99) transcripts in the consensus sequence;

Percent of high-quality isoforms (%): the percentage of high-quality transcripts in the consistent sequence.

Supplementary table S4 Statistics on the number of transcripts annotated by each database.

| **Annotated databases** | **Isoform Number** | **Ratio** |
| --- | --- | --- |
| COG | 16,431 | 40.18% |
| GO | 29,595 | 72.37% |
| KEGG | 18,160 | 44.41% |
| KOG | 24,800 | 60.65% |
| Pfam | 30,254 | 73.98% |
| Swiss-Prot | 24,153 | 59.06% |
| NR | 40,663 | 99.42% |
| All | 40,893 | |

Supplementary table S5 Distribution of annotated species that is statistics with KEGG annotation.

| Pathway | Pathway id | Gene number |
| --- | --- | --- |
| Carbon metabolism | ko01200 | 936 |
| Ribosome | ko03010 | 839 |
| Biosynthesis of amino acids | ko01230 | 715 |
| Protein processing in endoplasmic reticulum | ko04141 | 641 |
| Spliceosome | ko03040 | 609 |
| RNA transport | ko03013 | 487 |
| Oxidative phosphorylation | ko00190 | 398 |
| Starch and sucrose metabolism | ko00500 | 392 |
| Purine metabolism | ko00230 | 386 |
| Glycolysis / Gluconeogenesis | ko00010 | 369 |
| Endocytosis | ko04144 | 336 |
| Peroxisome | ko04146 | 323 |
| Ubiquitin mediated proteolysis | ko04120 | 319 |
| Amino sugar and nucleotide sugar metabolism | ko00520 | 312 |
| mRNA surveillance pathway | ko03015 | 305 |
| Pyruvate metabolism | ko00620 | 303 |
| RNA degradation | ko03018 | 288 |
| Glyoxylate and dicarboxylate metabolism | ko00630 | 285 |
| Ribosome biogenesis in eukaryotes | ko03008 | 281 |
| Pyrimidine metabolism | ko00240 | 261 |
| Phagosome | ko04145 | 251 |
| Cysteine and methionine metabolism | ko00270 | 250 |
| Carbon fixation in photosynthetic organisms | ko00710 | 245 |
| Aminoacyl-tRNA biosynthesis | ko00970 | 237 |
| Glutathione metabolism | ko00480 | 224 |
| Glycerophospholipid metabolism | ko00564 | 223 |
| Citrate cycle (TCA cycle) | ko00020 | 222 |
| Fatty acid metabolism | ko01212 | 215 |
| Proteasome | ko03050 | 212 |
| Glycine, serine and threonine metabolism | ko00260 | 211 |
| Alanine, aspartate and glutamate metabolism | ko00250 | 206 |
| Plant hormone signal transduction | ko04075 | 203 |
| 2-Oxocarboxylic acid metabolism | ko01210 | 197 |
| Plant-pathogen interaction | ko04626 | 190 |
| Nucleotide excision repair | ko03420 | 182 |
| Pentose phosphate pathway | ko00030 | 176 |
| Arginine and proline metabolism | ko00330 | 173 |
| Valine, leucine and isoleucine degradation | ko00280 | 170 |
| Fatty acid degradation | ko00071 | 167 |
| Fructose and mannose metabolism | ko00051 | 165 |
| Pentose and glucuronate interconversions | ko00040 | 157 |
| beta-Alanine metabolism | ko00410 | 153 |
| DNA replication | ko03030 | 148 |
| Glycerolipid metabolism | ko00561 | 143 |
| Phenylpropanoid biosynthesis | ko00940 | 143 |
| Ascorbate and aldarate metabolism | ko00053 | 141 |
| Inositol phosphate metabolism | ko00562 | 128 |
| Galactose metabolism | ko00052 | 122 |
| Phosphatidylinositol signaling system | ko04070 | 121 |
| Lysine degradation | ko00310 | 119 |
| Tryptophan metabolism | ko00380 | 117 |
| Mismatch repair | ko03430 | 117 |
| N-Glycan biosynthesis | ko00510 | 116 |
| Arginine biosynthesis | ko00220 | 115 |
| Propanoate metabolism | ko00640 | 115 |
| Fatty acid biosynthesis | ko00061 | 114 |
| Photosynthesis | ko00195 | 114 |
| Biosynthesis of unsaturated fatty acids | ko01040 | 113 |
| Phenylalanine, tyrosine and tryptophan biosynthesis | ko00400 | 111 |
| Terpenoid backbone biosynthesis | ko00900 | 109 |
| Basal transcription factors | ko03022 | 108 |
| Tyrosine metabolism | ko00350 | 107 |
| Homologous recombination | ko03440 | 107 |
| Phenylalanine metabolism | ko00360 | 102 |
| Protein export | ko03060 | 102 |
| Steroid biosynthesis | ko00100 | 96 |
| Cyanoamino acid metabolism | ko00460 | 96 |
| RNA polymerase | ko03020 | 94 |
| Circadian rhythm - plant | ko04712 | 91 |
| alpha-Linolenic acid metabolism | ko00592 | 86 |
| Regulation of autophagy | ko04140 | 84 |
| Butanoate metabolism | ko00650 | 83 |
| Porphyrin and chlorophyll metabolism | ko00860 | 83 |
| Sulfur metabolism | ko00920 | 80 |
| Ubiquinone and other terpenoid-quinone biosynthesis | ko00130 | 79 |
| Histidine metabolism | ko00340 | 74 |
| Ether lipid metabolism | ko00565 | 73 |
| SNARE interactions in vesicular transport | ko04130 | 73 |
| Valine, leucine and isoleucine biosynthesis | ko00290 | 72 |
| One carbon pool by folate | ko00670 | 69 |
| Thiamine metabolism | ko00730 | 66 |
| Pantothenate and CoA biosynthesis | ko00770 | 65 |
| Nitrogen metabolism | ko00910 | 64 |
| Base excision repair | ko03410 | 64 |
| Selenocompound metabolism | ko00450 | 62 |
| Tropane, piperidine and pyridine alkaloid biosynthesis | ko00960 | 60 |
| Nicotinate and nicotinamide metabolism | ko00760 | 59 |
| Sphingolipid metabolism | ko00600 | 57 |
| Isoquinoline alkaloid biosynthesis | ko00950 | 56 |
| Carotenoid biosynthesis | ko00906 | 54 |
| Glycosyl phosphate idylinositol(GPI)-anchor biosynthesis | ko00563 | 50 |
| Biotin metabolism | ko00780 | 47 |
| ABC transporters | ko02010 | 47 |
| Photosynthesis - antenna proteins | ko00196 | 44 |
| Other glycan degradation | ko00511 | 42 |
| Lysine biosynthesis | ko00300 | 39 |
| Arachidonic acid metabolism | ko00590 | 36 |
| AGE-RAGE signaling pathway in diabetic complications | ko04933 | 34 |
| Other types of O-glycan biosynthesis | ko00514 | 33 |
| Fatty acid elongation | ko00062 | 30 |
| Folate biosynthesis | ko00790 | 29 |
| Flavonoid biosynthesis | ko00941 | 29 |
| Sulfur relay system | ko04122 | 27 |
| Stilbenoid, diarylheptanoid and gingerol biosynthesis | ko00945 | 26 |
| Vitamin B6 metabolism | ko00750 | 25 |
| Synthesis and degradation of ketone bodies | ko00072 | 24 |
| Monobactam biosynthesis | ko00261 | 23 |
| Cutin, suberine and wax biosynthesis | ko00073 | 22 |
| C5-Branched dibasic acid metabolism | ko00660 | 22 |
| Glycosaminoglycan degradation | ko00531 | 21 |
| Non-homologous end-joining | ko03450 | 21 |
| Taurine and hypotaurine metabolism | ko00430 | 19 |
| Caffeine metabolism | ko00232 | 18 |
| Riboflavin metabolism | ko00740 | 18 |
| Sesquiterpenoid and triterpenoid biosynthesis | ko00909 | 16 |
| Linoleic acid metabolism | ko00591 | 15 |
| Limonene and pinene degradation | ko00903 | 9 |
| Glycosphingolipid biosynthesis - globo series | ko00603 | 8 |
| Glycosphingolipid biosynthesis - ganglio series | ko00604 | 8 |
| Lipoic acid metabolism | ko00785 | 7 |
| Zeatin biosynthesis | ko00908 | 7 |
| Betalain biosynthesis | ko00965 | 7 |
| Diterpenoid biosynthesis | ko00904 | 6 |
| Monoterpenoid biosynthesis | ko00902 | 4 |
| Vancomycin resistance | ko01502 | 4 |
| Brassinosteroid biosynthesis | ko00905 | 3 |
| Carbapenem biosynthesis | ko00332 | 1 |
| Glucosinolate biosynthesis | ko00966 | 1 |

Supplementary table S6 Statistical table for the evaluation of second-generation sequencing data of samples.

| **Samples** | **Read Number** | **Base Number** | **GC Content** | **%≥Q30** |
| --- | --- | --- | --- | --- |
| CK0-1 | 23,269,266 | 6,949,447,042 | 46.62 | 93.73 |
| CK0-2 | 18,547,194 | 5,535,737,084 | 46.32 | 93.95 |
| CK0-3 | 22,786,538 | 6,811,277,682 | 46.28 | 93.61 |
| T0-1 | 23,844,033 | 7,069,705,258 | 44.72 | 93.79 |
| T0-2 | 25,422,296 | 7,590,441,736 | 44.99 | 93.47 |
| T0-3 | 23,446,644 | 7,010,381,296 | 44.83 | 93.66 |
| CK1-1 | 24,665,345 | 7,370,451,930 | 44.62 | 93.7 |
| CK1-2 | 22,476,519 | 6,717,768,002 | 44.41 | 93.04 |
| CK1-3 | 19,046,779 | 5,695,472,434 | 44.38 | 93.86 |
| T1-1 | 19,607,636 | 5,845,862,330 | 43.77 | 94.03 |
| T1-2 | 18,498,377 | 5,523,759,116 | 43.61 | 93.49 |
| T1-3 | 22,150,580 | 6,621,407,556 | 43.53 | 93.46 |
| CK5-1 | 20,909,306 | 6,245,049,478 | 46.03 | 93.5 |
| CK5-2 | 21,882,375 | 6,537,472,472 | 47.2 | 94.18 |
| CK5-3 | 21,976,131 | 6,553,252,124 | 45.44 | 93.84 |
| T5-1 | 24,528,685 | 7,309,847,040 | 55.01 | 95.02 |
| T5-2 | 21,247,618 | 6,343,124,910 | 55.12 | 95.37 |
| T5-3 | 18,123,737 | 5,417,132,558 | 55.2 | 95.28 |
| CK8-1 | 21,485,486 | 6,416,649,742 | 45.87 | 93.72 |
| CK8-2 | 22,282,513 | 6,659,690,734 | 43.4 | 93.36 |
| CK8-3 | 20,419,762 | 6,102,163,540 | 43.54 | 93.64 |
| T8-1 | 20,949,365 | 6,269,978,652 | 49.52 | 93.57 |
| T8-2 | 25,645,184 | 7,666,796,990 | 49.7 | 93.84 |
| T8-3 | 21,403,595 | 6,400,318,106 | 49.13 | 93.83 |

Note: Samples: sample name of sample information sheet;

Read Number: Total number of pair-end Reads in Clean Data;

Base Number: Thetotal number of bases in Clean Data;

GC Content: The percentage of G and C bases of total bases in Clean Data;

≥Q30: The percentage of bases with a quality value of ≥30.

Supplementary table S7 Statistical table comparing second-generation sequencing data and third-generation non-redundant transcripts.

| **Sample** | **Total Reads** | **Uniquely mapped reads %** | **% of reads mapped to multiple loci** | **% of reads mapped to too many loci** |
| --- | --- | --- | --- | --- |
| CK0-1 | 23,269,266 | 30.94% | 31.16% | 21.58% |
| CK0-2 | 18,547,194 | 33.63% | 29.75% | 19.47% |
| CK0-3 | 22,786,538 | 33.64% | 28.96% | 19.09% |
| T0-1 | 23,844,033 | 36.51% | 28.63% | 13.63% |
| T0-2 | 25,422,296 | 37.51% | 27.87% | 12.56% |
| T0-3 | 23,446,644 | 37.82% | 27.86% | 11.74% |
| CK1-1 | 24,665,345 | 42.42% | 38.13% | 0.07% |
| CK1-2 | 22,476,519 | 42.02% | 37.29% | 0.05% |
| CK1-3 | 19,046,779 | 42.94% | 36.50% | 0.06% |
| T1-1 | 19,607,636 | 45.62% | 30.81% | 0.14% |
| T1-2 | 18,498,377 | 45.98% | 29.24% | 0.04% |
| T1-3 | 22,150,580 | 45.83% | 29.57% | 0.05% |
| CK5-1 | 20,909,306 | 49.40% | 24.23% | 0.14% |
| CK5-2 | 21,882,375 | 50.80% | 21.30% | 0.14% |
| CK5-3 | 21,976,131 | 48.55% | 25.10% | 0.18% |
| T5-1 | 24,528,685 | 47.63% | 11.78% | 0.22% |
| T5-2 | 21,247,618 | 47.70% | 11.84% | 0.23% |
| T5-3 | 18,123,737 | 47.40% | 11.47% | 0.22% |
| CK8-1 | 21,485,486 | 50.83% | 21.06% | 0.04% |
| CK8-2 | 22,282,513 | 47.52% | 25.76% | 0.05% |
| CK8-3 | 20,419,762 | 46.94% | 25.18% | 0.05% |
| T8-1 | 20,949,365 | 42.63% | 17.34% | 0.16% |
| T8-2 | 25,645,184 | 42.33% | 17.54% | 0.23% |
| T8-3 | 21,403,595 | 44.37% | 19.12% | 0.16% |

Note: Sample: the sample number;

Total Reads: the number of Clean Reads;

Uniquely mapped Reads%: the number of reads matched to the unique position of the transcript and its percentage in Clean Reads;

% of Reads mapped to multiple loci: the number of reads matched to multiple locations in the transcript and its percentage in Clean Reads;

% of Reads mapped to too many loci: the number of reads matched to many positions in the transcript and its percentage in Clean Reads.

Supplementary table S8 GO annotation functional classification of auxin related candidate genes.

| Gene ID | GO: annotation |
| --- | --- |
| *PB-BC37_transcript_892* | Molecular Function: DNA binding (GO:0003677);; Cellular Component: nucleus (GO:0005634);; Biological Process: regulation of transcription, DNA-templated (GO:0006355);; Biological Process: auxin-activated signaling pathway (GO:0009734); |
| *PB-BC37_transcript_930* | Molecular Function: DNA binding (GO:0003677);; Cellular Component: nucleus (GO:0005634);; Biological Process: regulation of transcription, DNA-templated (GO:0006355);; Biological Process: auxin-activated signaling pathway (GO:0009734);; |
| *PB-BC37_transcript_3548* | Cellular Component: nucleus (GO:0005634);; Biological Process: regulation of transcription, DNA-templated (GO:0006355);; Biological Process: auxin-activated signaling pathway (GO:0009734);; |
| *PB-BC37_transcript_4832* | Molecular Function: transcription factor activity, sequence-specific DNA binding (GO:0003700);; Cellular Component: nucleus (GO:0005634);; Biological Process: cell proliferation (GO:0008283);; Biological Process: response to auxin (GO:0009733);; Biological Process: unidimensional cell growth (GO:0009826);; Biological Process: shoot system morphogenesis (GO:0010016);; Molecular Function: protein homodimerization activity (GO:0042803);; Molecular Function: sequence-specific DNA binding (GO:0043565);; Biological Process: negative regulation of transcription, DNA-templated (GO:0045892);; Biological Process: root development (GO:0048364);; |
| *PB-BC37_transcript_5236* | Cellular Component: nucleus (GO:0005634);; Biological Process: regulation of transcription, DNA-templated (GO:0006355);; Biological Process: auxin-activated signaling pathway (GO:0009734);; |
| *PB-BC37_transcript_5985* | Cellular Component: nucleus (GO:0005634);; Biological Process: regulation of transcription, DNA-templated (GO:0006355);; Biological Process: auxin-activated signaling pathway (GO:0009734);; |
| *PB-BC37_transcript_6252* | Cellular Component: nucleus (GO:0005634);; Biological Process: regulation of transcription, DNA-templated (GO:0006355);; Biological Process: auxin-activated signaling pathway (GO:0009734);; |
| *PB-BC37_transcript_13039* | Cellular Component: nucleus (GO:0005634);; Biological Process: regulation of transcription, DNA-templated (GO:0006355);; Biological Process: auxin-activated signaling pathway (GO:0009734);; |
| *PB-BC37_transcript_16093* | Molecular Function: ATP binding (GO:0005524);; Cellular Component: plasma membrane (GO:0005886);; Biological Process: drug transmembrane transport (GO:0006855);; Biological Process: regulation of cell size (GO:0008361);; Molecular Function: xenobiotic-transporting ATPase activity (GO:0008559);; Cellular Component: plasmodesma (GO:0009506);; Biological Process: response to blue light (GO:0009637);; Biological Process: photomorphogenesis (GO:0009640);; Biological Process: auxin polar transport (GO:0009926);; Biological Process: positive gravitropism (GO:0009958);; Biological Process: auxin efflux (GO:0010315);; Molecular Function: auxin influx transmembrane transporter activity (GO:0010328);; Molecular Function: auxin efflux transmembrane transporter activity (GO:0010329);; Molecular Function: ferric-transporting ATPase activity (GO:0015408);; Biological Process: ferric iron transport (GO:0015682);; Cellular Component: integral component of membrane (GO:0016021);; Biological Process: xenobiotic transport (GO:0042908);; Biological Process: anthocyanin accumulation in tissues in response to UV light (GO:0043481);; Biological Process: stamen development (GO:0048443);; Biological Process: auxin influx (GO:0060919);; |
| *PB-BC37_transcript_23926* | Cellular Component: cell wall (GO:0005618);; Cellular Component: nucleus (GO:0005634);; Cellular Component: cytosol (GO:0005829);; Cellular Component: plasma membrane (GO:0005886);; Biological Process: response to auxin (GO:0009733);; Biological Process: xylem and phloem pattern formation (GO:0010051);; Biological Process: vegetative to reproductive phase transition of meristem (GO:0010228);; Biological Process: SCF complex assembly (GO:0010265);; Biological Process: protein ubiquitination (GO:0016567);; |
| *PB-BC37_transcript_27545* | Biological Process: auxin-activated signaling pathway (GO:0009734);; Cellular Component: integral component of membrane (GO:0016021);; Biological Process: transmembrane transport (GO:0055085);; |
| *PB-BC37_transcript_35225* | Molecular Function: DNA binding (GO:0003677);; Cellular Component: nucleus (GO:0005634);; Biological Process: regulation of transcription, DNA-templated (GO:0006355);; Biological Process: auxin-activated signaling pathway (GO:0009734);; |
| *PB-BC37_transcript_41880* | Molecular Function: GTPase activator activity (GO:0005096);; Cellular Component: cytosol (GO:0005829);; Cellular Component: plasma membrane (GO:0005886);; Biological Process: endocytosis (GO:0006897);; Biological Process: response to auxin (GO:0009733);; Biological Process: leaf morphogenesis (GO:0009965);; Biological Process: xylem and phloem pattern formation (GO:0010051);; Biological Process: phloem or xylem histogenesis (GO:0010087);; Cellular Component: trans-Golgi network transport vesicle (GO:0030140);; Molecular Function: phosphatidylinositol binding (GO:0035091);; Biological Process: positive regulation of GTPase activity (GO:0043547);; |
| *PB-BC37_transcript_46433* | Cellular Component: nucleus (GO:0005634);; Biological Process: regulation of transcription, DNA-templated (GO:0006355);; Biological Process: auxin-activated signaling pathway (GO:0009734);; |
| *PB-BC37_transcript_46530* | Cellular Component: nucleus (GO:0005634);; Biological Process: regulation of transcription, DNA-templated (GO:0006355);; Biological Process: auxin-activated signaling pathway (GO:0009734);; |
| *PB-BC37_transcript_46773* | Molecular Function: DNA binding (GO:0003677);; Cellular Component: nucleus (GO:0005634);; Biological Process: regulation of transcription, DNA-templated (GO:0006355);; Biological Process: auxin-activated signaling pathway (GO:0009734);; |
| *PB-BC37_transcript_57575* | Molecular Function: DNA binding (GO:0003677);; Cellular Component: nucleus (GO:0005634);; Biological Process: regulation of transcription, DNA-templated (GO:0006355);; Biological Process: auxin-activated signaling pathway (GO:0009734);; |
| *PB-BC37_transcript_58353* | Molecular Function: DNA binding (GO:0003677);; Cellular Component: nucleus (GO:0005634);; Biological Process: regulation of transcription, DNA-templated (GO:0006355);; Biological Process: auxin-activated signaling pathway (GO:0009734);; |

Supplementary table S9 Functional annotation analysis of related genes connected with hub genes.

| Gene ID | KEGG annotation | KEGG pathway | Pfam annotation | Swissprot annotation | eggnog class | NR annotation |
| --- | --- | --- | --- | --- | --- | --- |
| *PB-BC37_transcript_892* | K14486\|0\|bvg:104900616\|K14486 auxin response factor \| (RefSeq) auxin response factor 3 | Plant hormone signal transduction (ko04075) | Auxin response factor;; B3 DNA binding domain | Auxin response factor 3 GN=T1B8.30 OS=Arabidopsis thaliana (Mouse-ear cress) PE=1 SV=2 | Transcription | auxin response factor 3-like [Chenopodium quinoa] |
| *PB-BC37_transcript_930* | K14486\|0\|bvg:104900616\|K14486 auxin response factor \| (RefSeq) auxin response factor 3 | Plant hormone signal transduction (ko04075) | Auxin response factor;; B3 DNA binding domain | Auxin response factor 3 GN=T1B8.30 OS=Arabidopsis thaliana (Mouse-ear cress) PE=1 SV=2 | Transcription | PREDICTED: auxin response factor 3 isoform X2 [Beta vulgaris subsp. vulgaris] |
| *PB-BC37_transcript_5985* | K14484\|3.46815e-98\|bvg:104904635\|K14484 auxin-responsive protein IAA \| (RefSeq) auxin-responsive protein IAA1 | Plant hormone signal transduction (ko04075) | AUX/IAA family | Auxin-responsive protein IAA16 GN=IAA16 OS=Arabidopsis thaliana (Mouse-ear cress) PE=1 SV=1 | Function unknown | auxin-responsive protein IAA1-like [Chenopodium quinoa] |
| *PB-BC37_transcript_13039* | K14484\|5.35824e-115\|bvg:104894591\|K14484 auxin-responsive protein IAA \| (RefSeq) auxin-induced protein 22D | Plant hormone signal transduction (ko04075) | AUX/IAA family | Auxin-induced protein 22D GN=AUX22D OS=Vigna radiata var. radiata (Mung bean) PE=2 SV=1 | Transcription | PREDICTED: auxin-induced protein 22D [Beta vulgaris subsp. vulgaris] |
| *PB-BC37_transcript_27545* | K13947\|0\|gra:105785713\|K13947 auxin efflux carrier family \| (RefSeq) auxin efflux carrier component 4 | -- | Membrane transport protein | Auxin efflux carrier component 3 GN=PIN3 OS=Arabidopsis thaliana (Mouse-ear cress) PE=1 SV=1 | Intracellular trafficking, secretion, and vesicular transport | auxin efflux carrier component 3-like isoform X1 [Chenopodium quinoa] |
| *PB-BC37_transcript_35225* | K14486\|0\|bvg:104908192\|K14486 auxin response factor \| (RefSeq) auxin response factor 9 | Plant hormone signal transduction (ko04075) | Auxin response factor;; B3 DNA binding domain;; AUX/IAA family | Auxin response factor 9 GN=ARF9 OS=Arabidopsis thaliana (Mouse-ear cress) PE=1 SV=1 | Transcription | auxin response factor 9-like [Spinacia oleracea] |

**
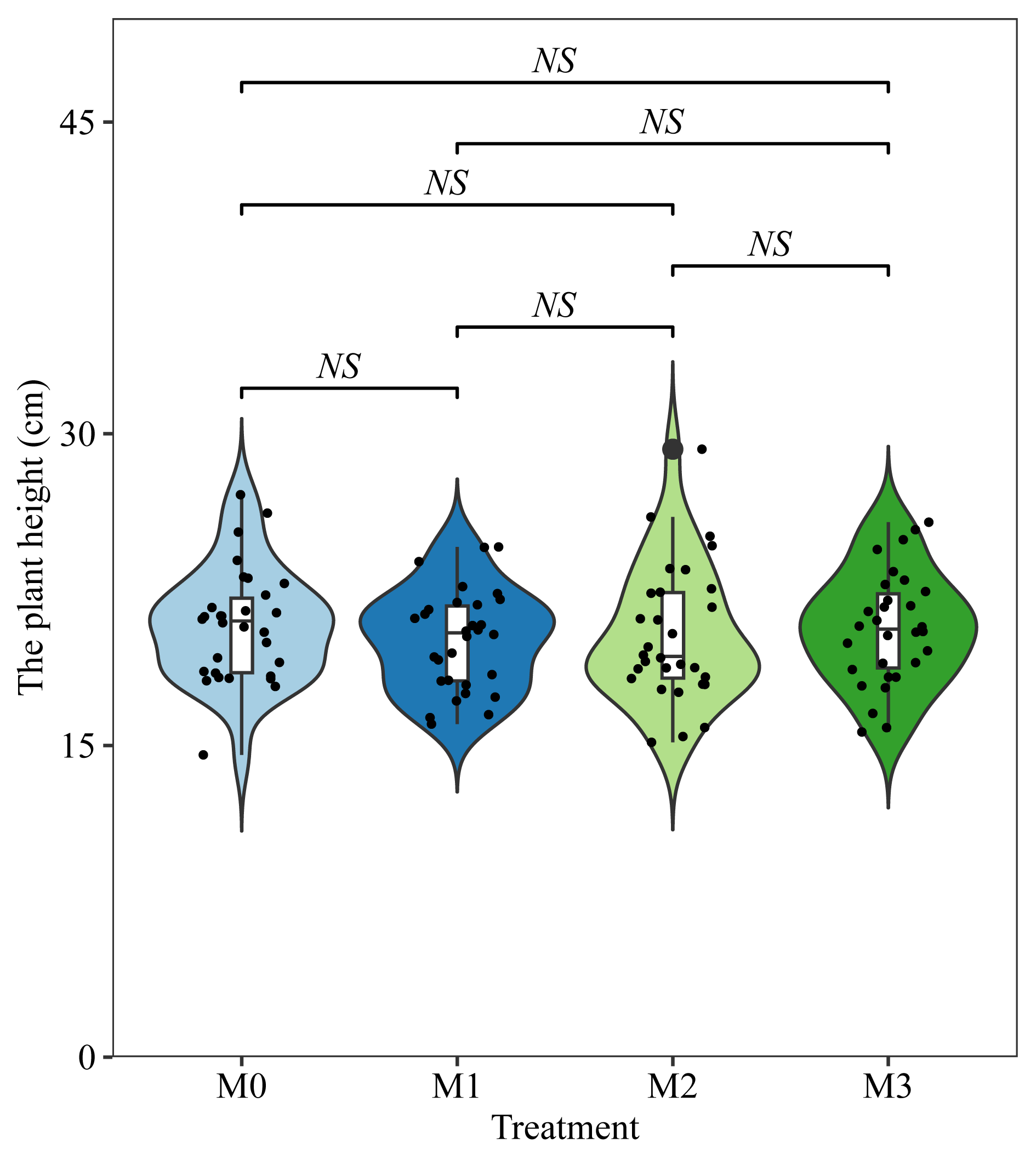
**

**Supplementary figure S1** The basic situation of the growth of experimental materials.

Note: *NS* means no significant difference

**
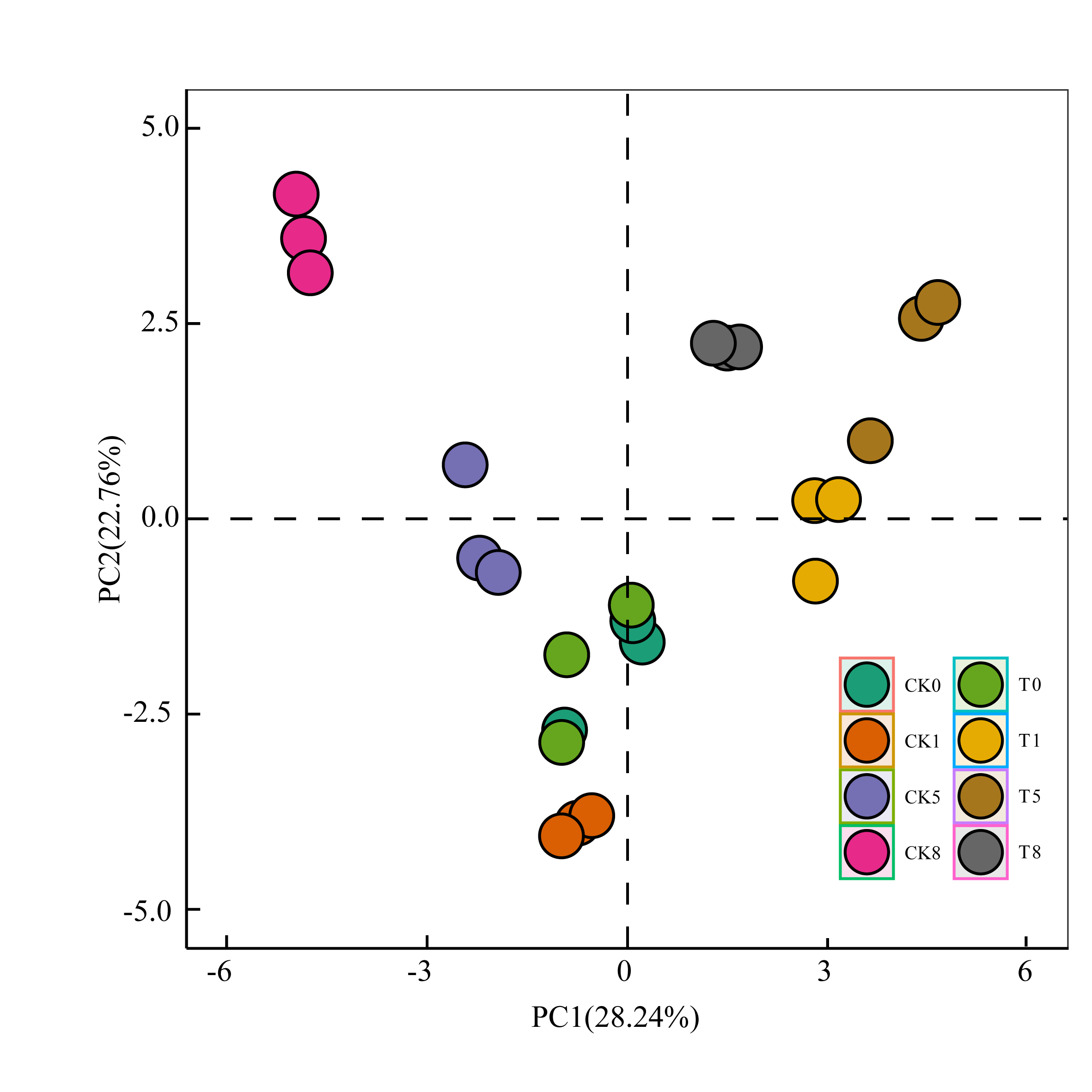
**

**Supplementary figure S2** Principal component analysis (PCA) based on metabolite abundance in samples.

**
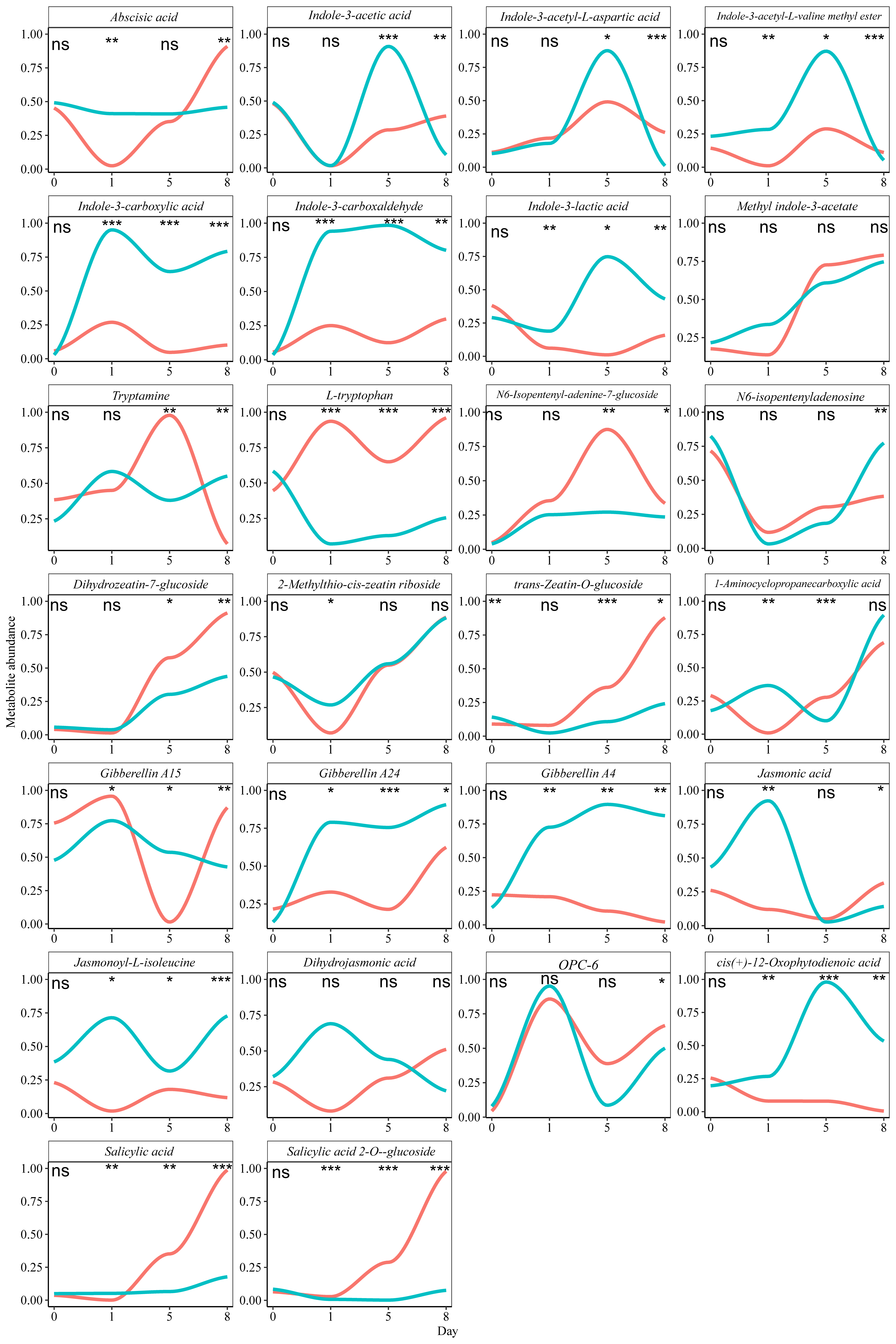
Supplementary figure S3** Analysis of metabolite accumulation patterns.

Note：The red and blue lines represent M1 and M0 treatment, respectively.

**
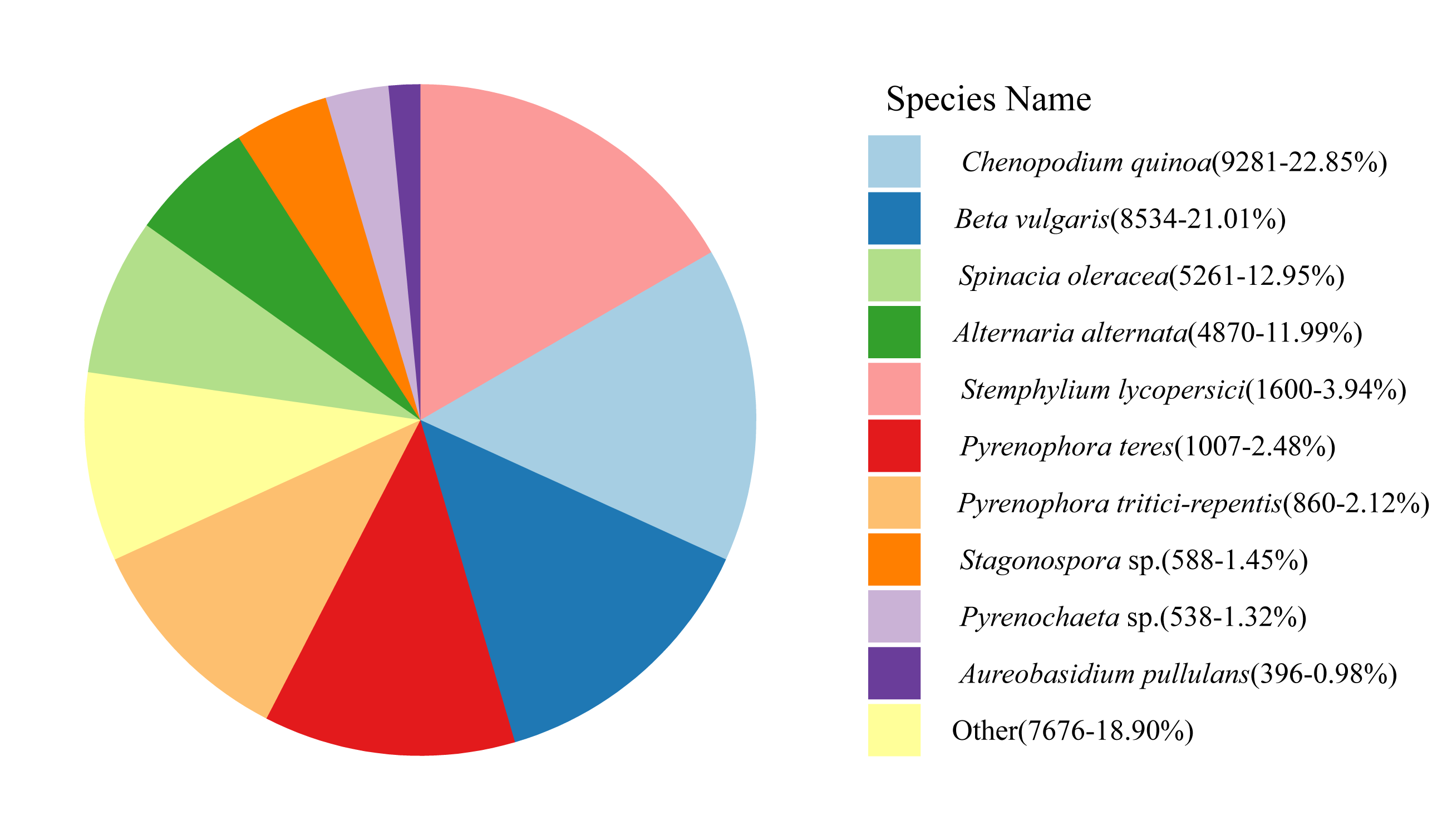
**

**Supplementary figure S4** Distribution of annotated species that is statistics with NR annotation.
